# Supplementary material for: The Analysis of Embryoid Body Formation and Its Role in Retinal Organoid Development
Source: Int J Mol Sci. 2024 Jan 24;25(3):1444. doi: 10.3390/ijms25031444 (PMC10855324; doi:10.3390/ijms25031444)
Supplement: Supplementary file 1 [file ijms-25-01444-s001.zip › ijms-2801365-supplementary.pdf]

Additional File 1

The analysis of embryoid body formation and its role in retinal organoid development

Andrea Heredero Berzal, Ellie L. Wagstaff, Anneloor L. M. A. ten Asbroek, Jacoline B. ten Brink, Arthur A. Bergen, Camiel J. F. Boon.

Figure S1 : Brightfield images of neurospheres generated under the clumps protocol (CP) and single cells protocol (SCP).

Figure S2: Expression of primitive endoderm marker GATA4 and the primitive ectoderm marker SOX2 in embryoid bodies (EBs) on day 4 and 7.

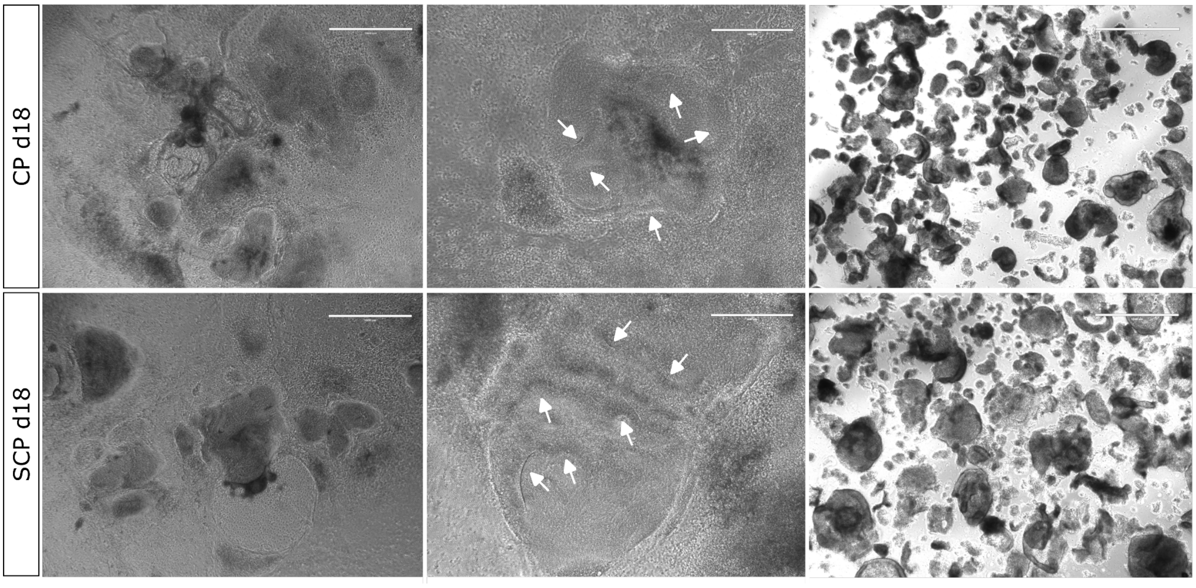

Figure S1 . Representative brightfield images of neurospheres generated under the clumps protocol (CP) and single cells protocol (SCP) on day 18. (Left) representative image of the neurospheres, scale bars = 1000 μm. (Middle) detail of the neurospheres in higher magnification, and highlighted with white arrows the eye-field primordial clusters, scale bars = 400 μm. (Right) Floating eye-field primordial clusters after scraping step on day 18. This material remains floating upon differentiation, scale bars = 1000 μm.

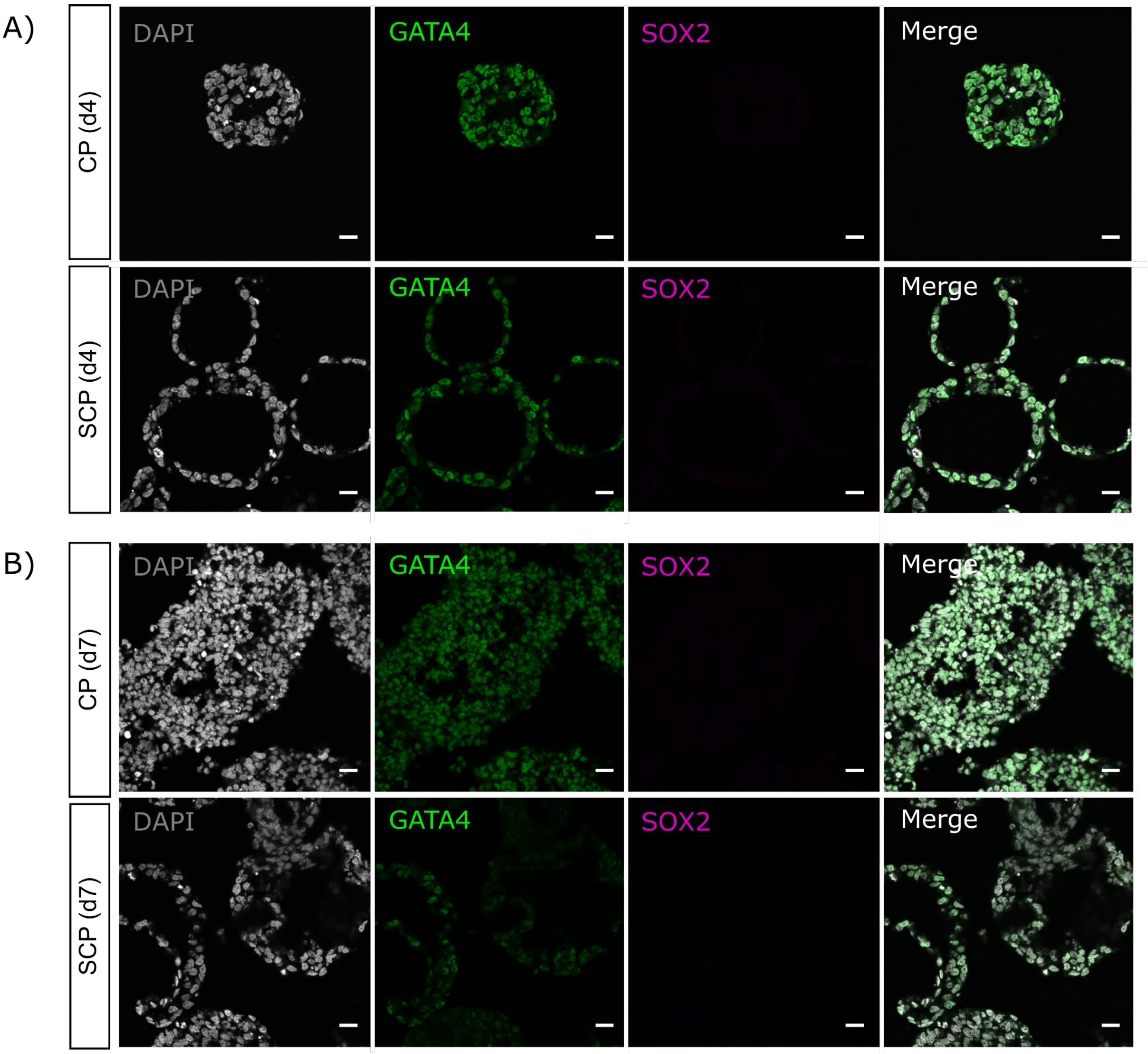

Figure S2. A) Representative images of immunohistochemistry sections of embryoid bodies (EBs) on day 4 (for both protocols) presenting DAPI-stained nuclei (in gray), the primitive endoderm marker GATA4 (in green) and the primitive ectoderm marker SOX2 (in magenta). B) Representative images of immunohistochemistry sections of EBs on day 7 (for both protocols) presenting GATA4 (green) and SOX2 (magenta). Scale bars = 20 μm for both protocols on day 4 and 7.
